# Rheology and microstructure effects of waste spent coffee grounds in modifying asphalt binder

**DOI:** 10.1007/s44242-022-00004-0

**Published:** 2023-02-01

**Authors:** Mingjun Xie, Linglin Xu, Kai Wu, Yutong Wen, Hongmi Jiang, Zhengwu Jiang

**Affiliations:** 1 Key Laboratory of Advanced Civil Engineering Materials of Ministry of Education, School of Materials Science and Engineering, Tongji University, Shanghai, 201804 China; 2 Shanghai Pinghe Bilingua,School, Shanghai, 201206 China

**Keywords:** Spent coffee grounds, Asphalt binder, Bio-waste management, Rheology properties, Microstructure, 废弃咖啡渣, 沥青粘结剂, 生物废弃物管理, 流变学特性, 微观结构

## Abstract

Herein the biowaste by-product spent coffee grounds (SCGs) from coffee industry were incorporated into asphalt binders for performance enhancement. From the analysis of Fourier transform infrared spectroscopy (FTIR), differential scanning calorimetry (DSC), dynamic shear rheometer (DSR), and Brookfield viscosity rheometer, it is confirmed that SCGs have potential prospects as bio-waste modifiers in the application of sustainable pavements. Results demonstrated that the modification process was mainly based on physical reinforcement. Compared with that of the neat asphalt, the shearing stress-resistant ability and high-temperature performance of the SCGs modified binders with the appropriate addition presented a bit of improvement; whereas the binders with 1% and 3% SCGs exhibited remarkably enhanced low-temperature stability. However, notable weaknesses of practical performance were shown for the binder with excessive content of SCGs, indicating the necessity of proportion selecting before application.

## Abstract (zh)

本文将咖啡萃取后得到的固体废弃物咖啡渣(Spent coffee grounds, SCGs)应用于沥青材料改性中，旨在实现生物基固废材料处理和沥青路面性能提升的双重目的。通过傅里叶红外光谱(FTIR)、差示扫描量热仪(DSC)、动态剪切流变仪(DSR)和布氏黏度计等技术，证实了咖啡渣在路面材料可持续化发展中具有潜在的应用与推广前景。试验结果表明，咖啡渣改性过程主要基于物理增强作用；与基质沥青相比，合理掺量的咖啡渣改性沥青具有理想的高温稳定性和良好的抗永久变形能力；同时1%和3%掺量的咖啡渣引入有利于增强基质沥青的低温性能。然而，过多咖啡渣的加入可能会对改性沥青的流变性能产生负面影响，这表明改性时应选择合理掺量的咖啡渣改性剂以满足实际性能提升需求。

**Graphical Abstract:**

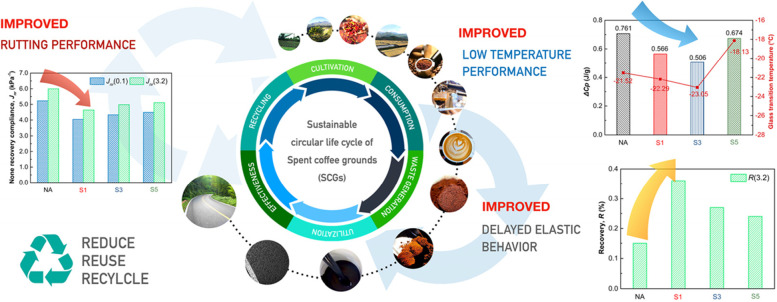

## Introduction

Coffee has been the second most traded product that shows essential impacts on the development of agriculture and global economy [1–3]. According to International Coffee Organization (ICO), although affected by COVID-19, about 10.1 million tons of coffee has been consumed from 2019 to 2020, and this data is estimated to climb up by 0.3% compared with that of 2018/2019 [4]. Spent coffee grounds (SCGs) are the by-product collected after coffee brewing. Generally they are discarded directly as waste for landfills with no added value. As one type of organic solid waste, it is worth remarking that more potential risks of SCGs are exposure to exert toxic influences on the environment, which attributes to their higher contents of organic chemical compounds—i.e. caffeine, tannins, polyphenols—diverse from other waste materials [1, 5, 6]. Recently, Shanghai has been confirmed as the metropolis possessing the most number of coffee shops in the worldwide [7, 8]. With more than 7000 coffee shops located in [9], there is no doubt that a huge amount of SCGs is generated in Shanghai everyday. Meanwhile, this data even accounts for 6 million tons of SCGs generated annually worldwide [10], thus contributing to serious consideration for waste management.

Lots of efforts have been made to promote the additional value of SCGs and make rational use of them, which can be illustrated with the examples of the purification of high-value bioactive compounds [11–14], activated carbon [15], or as the absorbents for heavy metals removal [16], energy fuels [17, 18], and oil extraction [19, 20], etc. Regardless of rather fewer SCGs consumed, however, a complicated reutilizing procedure leads to an increased cost budget, which restricts further application on a large scale.

Construction industry in civil engineering and transportation accounts for a huge percentage of carbon emissions of the world. More importantly, severe climate problems imply the urgency of sustainable development for construction materials to mitigate global warming [21, 22]. For these reasons, the disposal of solid waste with less carbon footprint draws more attention in both academia and engineering applications. It cannot only achieve the adequate utilization of solid waste and reduce the environmental repercussions but even have certain improvements in performing of the final products [23–26]. In recent reports, SCGs were incorporated into cementitious materials and other binding systems for building and road constructions. Eliche-Quesada et al. [27, 28] found that the modified clay bricks with 3% SCGs exhibited acceptable compressive strength and better insulating thermal capacity as compared to the control samples. Besides, several reports [29, 30] demonstrated that the thermal conductivities of bricks were in obvious decline owing to the remarkably increasing addition of SCGs, which indicates their promising application in thermal behavior regulation as the thermal insulator. The increase of SCGs content may also contribute to the decreased bulk density; whereas the enlarged porosity would also lead to a reduction in compressive strength [31], suggesting the importance of appropriate dosage for application.

As for pavement construction, several works of SCGs were conducted to determine their potential viability in road embankment applications. Arulrajah et al. [32] found that the adoption of SCGs was hard to resist high traffic loadings as structural filling materials of embankments, this hence provides the possibility for SCGs to be used as non-structural filling materials in road embankments. To cope with the drawbacks, several additional efforts proposed to incorporate SCGs into geopolymers along with mineral admixtures, including fly ash [33], slag and fly ash [34], hydrated lime and Portland cement [35], etc., which ensures the enhanced stabilization and appropriate mechanical properties as filling materials of road subgrade or structural embankment. Asphalt is a common composition derived from the petroleum refining process which is widely applied to the pavement industry; however, barely has the interaction mechanism between SCGs and asphalt binder been studied in previous reports. Owing to the main components of hemicellulose, lignin, amorphous cellulose and oil fraction [36], it is expected that the performance of asphalt binders can be effectively improved by the incorporation of SCGs. On the whole, it is of great value to determine and summarize the immediate impact of SCGs on the physicochemical and practical properties of asphalt binders.

**Fig. 1:**
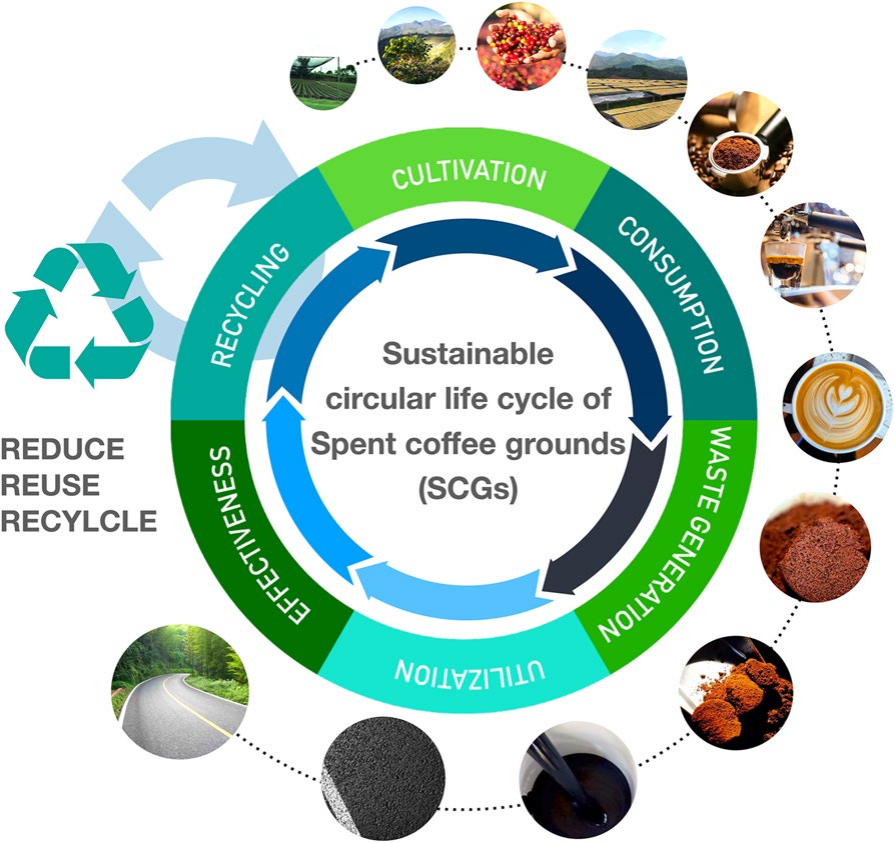
Schematic diagram of the sustainable life cycle of SCGs from generating to reutilizing and recycling

Herein, we proposed an effective utilization approach for biowaste by-product SCGs to achieve the dual purpose of bio-waste management and characterizing properties enhancement for asphalt binders. As shown in Fig. 1, the circular cycle follows a sustainably developing process starting from the waste generation to reutilizing, and recycling, eventually accomplishing a more environmental life cycle of SCGs. For this purpose, thereby, SCGs were adopted as the modifier incorporat into asphalt binder. Fourier transform infrared spectroscopy (FTIR) and scanning electron microscopy (SEM) was conducted to clarify the chemical composition variation and micro-morphology features of asphalt binder samples with SCGs. Different dosage was untaken to investigate the rheological characteristic in terms of dynamic shear rheometer (DSR), also the low-temperature stability of asphalt binders was studied using differential scanning calorimetry (DSC) for the asphalt binders. This work provides a promising insight into the reutilization of organic solid waste in the paving asphalt industry.

## Material and methods

### Materials and preparation

**Fig. 2:**
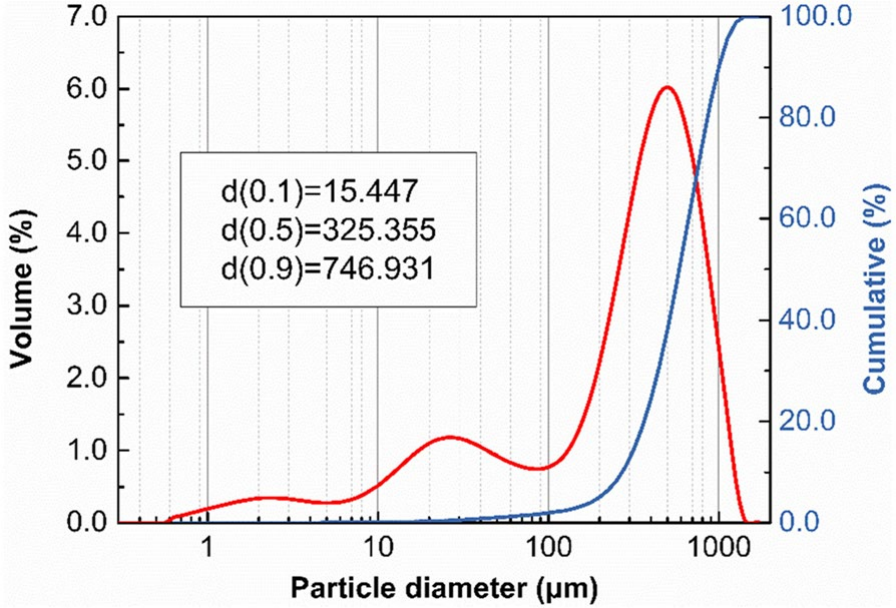
The median particle size distribution of SCGs

In ths work, asphalt with the 60/80 penetration grade was used as the control binder. Spent coffee grounds (SCGs), blended from the Asia Pacific and Latin America coffee belts, were acquired from a local commercial coffee shop freely. Before being used as the asphalt binder modifier, they have been oven-dried at 105 °C for 24 h to remove the residual evaporated water. Also, the median particle size was evaluated by laser particle sizes distribution analyzer (Fig. 2).

**Fig. 3:**
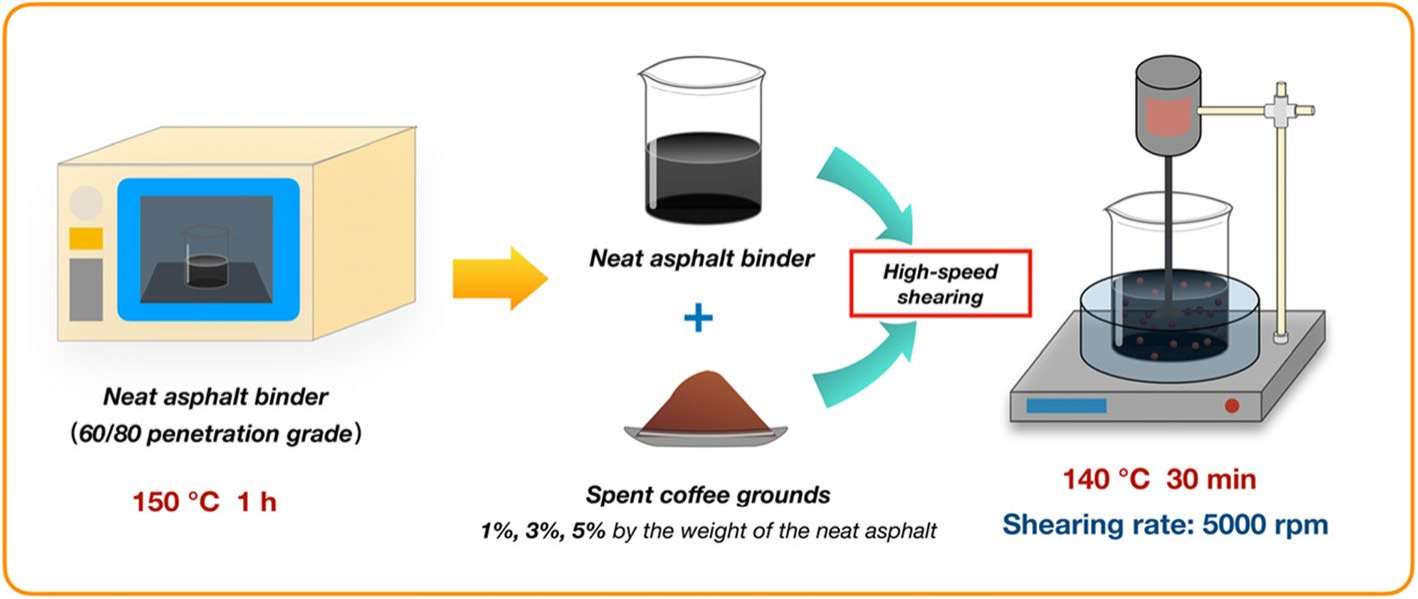
Schematic diagram of the preparation process of SCGs modified asphalt

The SCGs modified asphalt binders were fabricated by using a high-speed shearing and dispersing and emulsifying machine to attain a homogenous state, of which the modifying process can be summed up in Fig. 3. The modified binders with 1%, 3% and 5% SCGs by the weight of asphalt were marked as S1, S3, S5, respectively; while the neat control binder was directly listed as NA.

### Experimental methods

#### Rheology analysis

The rotational viscosity was tested using a Brookfield viscosity rheometer (Brookfield, DV2TRVTJ0, USA) to investigate the viscosity-temperature characteristics of binders from 120 °C ~ 200 °C in this study, with the test methods complied with ASTM D4402/D4402M-15. In accordance with ASTM D7175–15, furthermore, the rheological properties of asphalt binders were determined using a dynamic shear rheometer (DSR, SmartPave 92, Anton Paar GmbH, Austria) within intermediate and high-temperature ranges. The temperature sweep mode of DSR was conducted on all asphalt binders from 30 °C to 90 °C with a shearing angular frequency of 10 rad/s and a heating rate of 1 °C/min. The complex shear modulus (G*), phase angle (*δ*) and rutting factor (G*/sinδ) can be obtained to reveal the temperature stability of asphalt binders. Meanwhile, the rheological performance was also determined from the frequency sweep tests using a small strain amplitude from 100 Hz to 0.01 Hz at a constant temperature of 35 °C, of which the results illustrate the undamaged stiffness of binders within the linear viscoelastic (LVE) region.

The multiple stress creep recovery (MSCR) test is one of the most effective approaches to evaluate the rutting resistance properties of asphalt binders using DSR instruments. In accordance with AASHTO T 350–14, Two creep stress levels (0.1 kPa and 3.2 kPa) were applied to the binder specimens for 1 s, then followed by a recovery duration of 9 s. The loading recovery procedure would be repeated for 10 times in each stress level at a temperature of 64 °C. In addition, two parameters of non-recoverable compliance (*J*_*nr*_) values and recovery (*R*) values were calculated and analyzed to evaluate the permanent deformation resistance and delayed elastic behavior of binders respectively.

#### Characterization

The chemical structure and functional groups of asphalt binder samples and SCGs were characterized by Attenuated Total Reflection Fourier transform infrared spectroscopy (ATR-FTIR, Thermo Scientific Nicolet iS5, USA) with the scanning rate of 4000 cm^− 1^ to 500 cm^− 1^. In order to investigate the thermal behavior and low-temperature stability of asphalt binders, differential scanning calorimetry analysis (DSC 2500, TA instruments, USA) was measured from − 50 °C to 150 °C with a scanning rate of 10 °C/min under the nitrogen atmosphere, where the glass transition temperature (*T*_*g*_) and endothermic enthalpy (*△cp*) can be obtained from the analysis curves. It is mentioned that the thermal history of different samples has been eliminated before testing. Otherwise, a scanning electron microscope (SEM, ZEISS Gemini 300, Germany) has been conducted in the secondary electron mode to observe the microstructure and surface morphology features of tested modified binders along with SCGs (gold-sprayed) with an accelerated voltage of 15 kV.

## Results and discussion

### Chemical structure

**Fig. 4:**
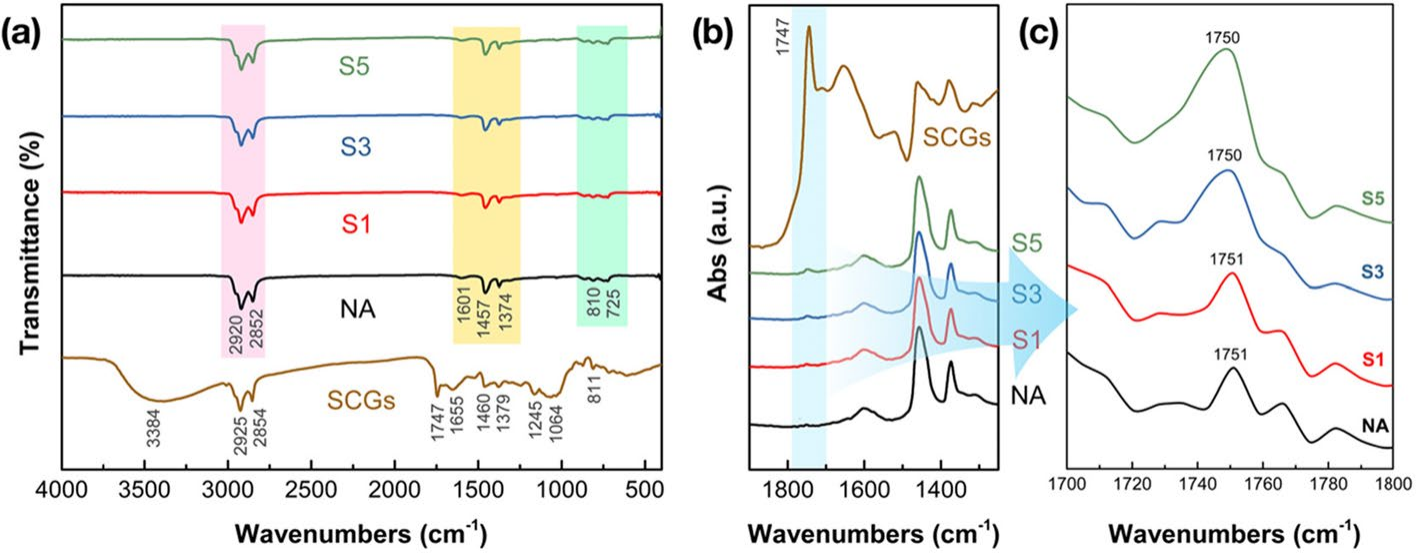
FTIR results of modified asphalt binders, NA neat binder and SCGs, **a** wavenumber from 4000 cm^− 1^ ~ 500 cm^− 1^; **b** specific wavenumber range from 1250 cm^− 1^ ~ 1900 cm^− 1^; (**c**) specific wavenumber range from 1700 cm^− 1^ ~ 1800 cm^− 1^

FTIR analysis was carried out to illustrate the chemical structure of asphalt binders and SCGs, which is presented in Fig. 4. The identified chemical functional groups of SCGs are shown in Fig. 4(a). Specifically, characteristic peaks observed at 2925 cm^− 1^ and 2854 cm^− 1^ can be attributed to asymmetric and symmetric stretching of C–H in –CH_3_ and –CH_2_ groups of caffeine molecule, respectively. Meanwhile, the characteristic peaks at 1460 cm^− 1^, 1379 cm^− 1^ and 811 cm^− 1^ can be attributed to the β-linkage of cellulose [37]. Furthermore, the characteristic peak at 1747 cm^− 1^ corresponds to the –C=O stretching vibration of O=C–O from aliphatic ester or triglycerides [38]. The vibration band at 1510 cm^− 1^ is related to C=C stretching of the aromatic ring in lignin or lipids, whereas the peaks at 1064 cm^− 1^ and 1245 cm^− 1^ are corresponding to the C–O stretching vibrations from monosaccharides as well as diverse acids molecules, including chlorogenic acid, caffeic acid and coumaric acid [39].

Almost no difference can be found from Fig. 4(a) when comparing the function groups of NA binder along with the modified asphalt binders, since the rather low dosage level is not enough to initiate an evident transformation in FTIR results. Indeed, all the characteristic peaks of NA binder are exhibited in the modified samples, where the bands at ~ 2920 cm^− 1^ and ~ 2852 cm^− 1^ are assigned to asymmetric and symmetric stretching of C–H. The vibrations at ~ 1601 cm^− 1^ (C=C stretching vibration of aromatic ring), 1457 cm^− 1^ (−CH_2_ scissoring vibration), and 1374 cm^− 1^ (−CH_3_ in-plane bending vibration) can also be identified when compared with the control sample. This finding presents an indication that the modifying process mainly results from physical effects.

To further determine the variation of chemical bonds after being modified by SCGs, the characteristic absorbance spectra are presented in Fig. 4(b) from 1250 cm^− 1^ to 1900 cm^− 1^ and Fig. 4(c) from 1700 cm^− 1^ to 1800 cm^− 1^. In contrast with NA binder, it can be noticed that the carbonyl absorption peak at 1747 cm^− 1^ (originated from –C=O stretching vibration) is simultaneously improved as the dosage of SCGs increases. This can be attributed to the chemical interaction between the neat asphalt and chemical compounds in SCGs, primarily including aliphatic ester or triglycerides.

### Rotational viscosity-temperature characteristics

**Fig. 5:**
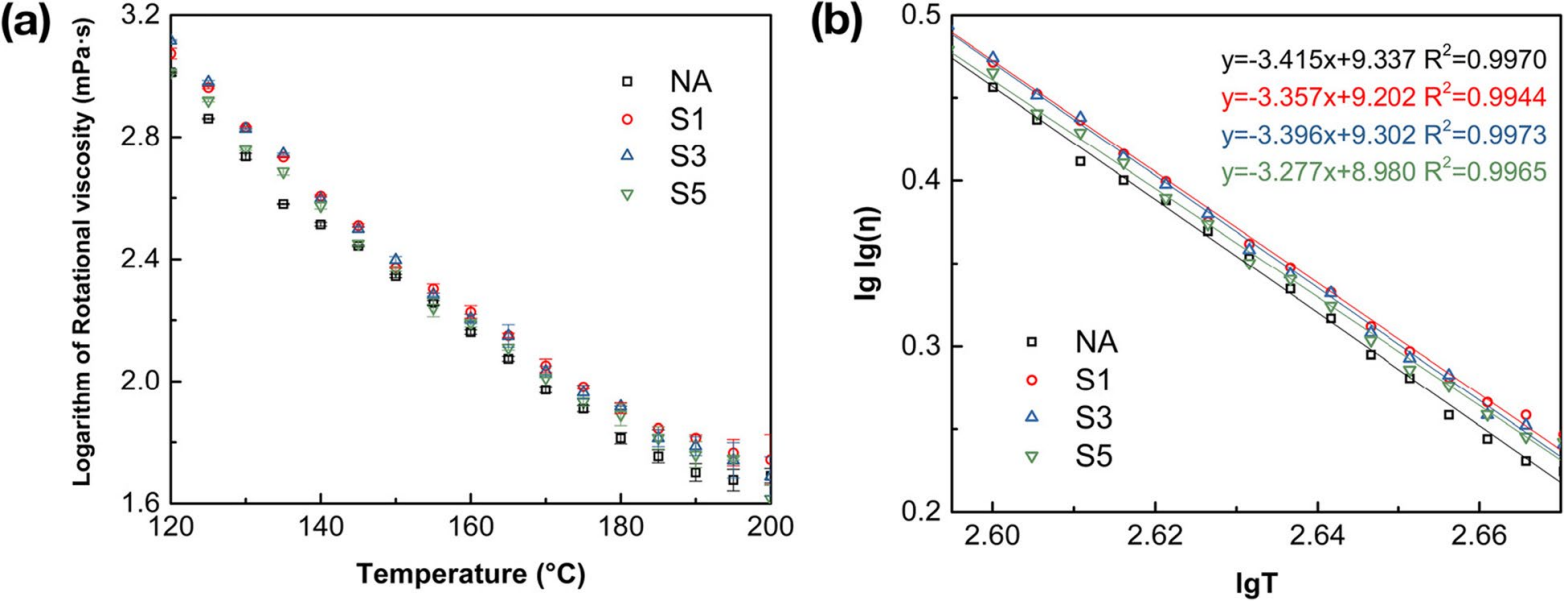
Rotational viscosity-temperature characteristics of the asphalt, **a** viscosity-temperature chart under the range of 120 °C ~ 200 °C; **b** fitted curves based on the Saal model

The viscosity-temperature relationship is an important indicator to evaluate the high-temperature performance of asphalt binders. Figure 5(a) demonstrates the results of Brookfield rotational viscosity-temperature curves under the range of 120 °C ~ 200 °C. It is evident that the incorporation of SCGs contributes to the increased viscosity, and the rotational viscosity of SCGs modified binders met the requirement of workability and mixability in operation complied with ASTM D6373, which should be no more than 3000 mPa·s at a temperature of 135 °C. Among this temperature range, the asphalt binders with 1% and 3% SCGs exhibit higher rotational viscosity compared to others. This is because the stiffening of binder increased with the addition of SCGs, thereby illustrating the modified binders display enhanced high-temperature stability and viscosity-temperature performance by the incorporation of SCGs. Nevertheless, the improved viscosity-temperature characteristic is restricted by the increasing addition, although the modified binder with 5% SCGs shows slightly higher viscosity than the control (NA). This could be explained by the fact that although SCGs are able to enhance the rotational viscosity of modified binders, the increased organic compounds (i.e. the oil fractions) of SCGs mainly dominate the binder easy to flow, thus the rotational viscosity of 5% SCGs modified asphalt is reduced as the dose increases.

The Saal model from ASTM-D2493/D2493M-16 has also been adopted to analyze the viscosity-temperature characteristic of different binders, which is listed in the following Eq. (1).

1 $$lglg\left(\eta \right)=n- mlgT$$

Where *η* represents the viscosity of asphalt binder (mPa·s), and *T* is the tested temperature (K). *m* and *n* is the regression coefficient, where *m* stands for the regression intercept and *n* represents the regression slop, respectively.

*m* and *n* values obtained from regression analysis are presented in Fig. 5(b). *m* value is an important parameter to evaluate the viscosity-temperature susceptibility of binders. It can be observed from Fig. 5(b) that the viscosity-temperature susceptibility parameter *m* decreases from 3.415 for the neat binder to 3.357, 3.396, 3.277, for S1, S3, S5, respectively. The decreased *m* value contributes to lower temperature sensitivity [40]. The results demonstrated that the SCG modifier has an optimal impact on the reduction of viscosity-temperature susceptibility, and the m value of binder decreases with the increased content of SCGs, which indicated that the adoption of SCGs enhanced the temperature stability of asphalt binder.

### Undamaged stiffness properties

**Fig. 6:**
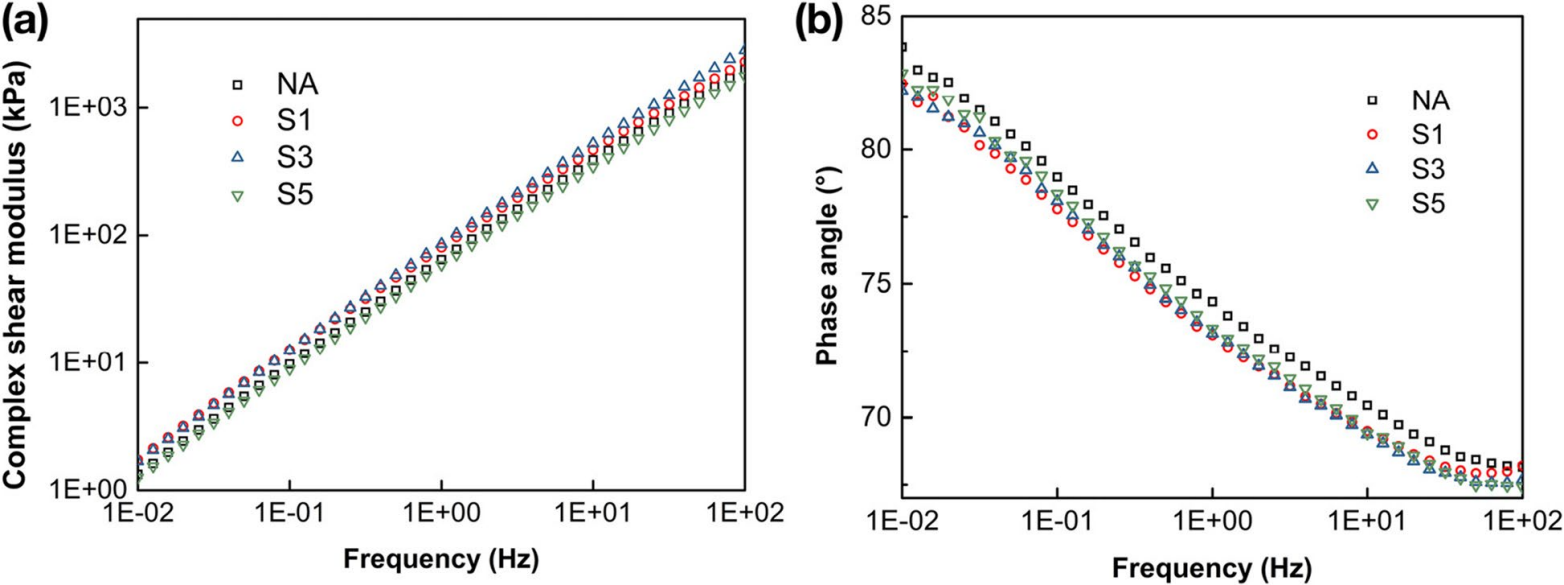
The frequency sweep results of SCGs modified binders and NA binder, **a** complex shear modulus; **b** phase angle

**Fig. 7:**
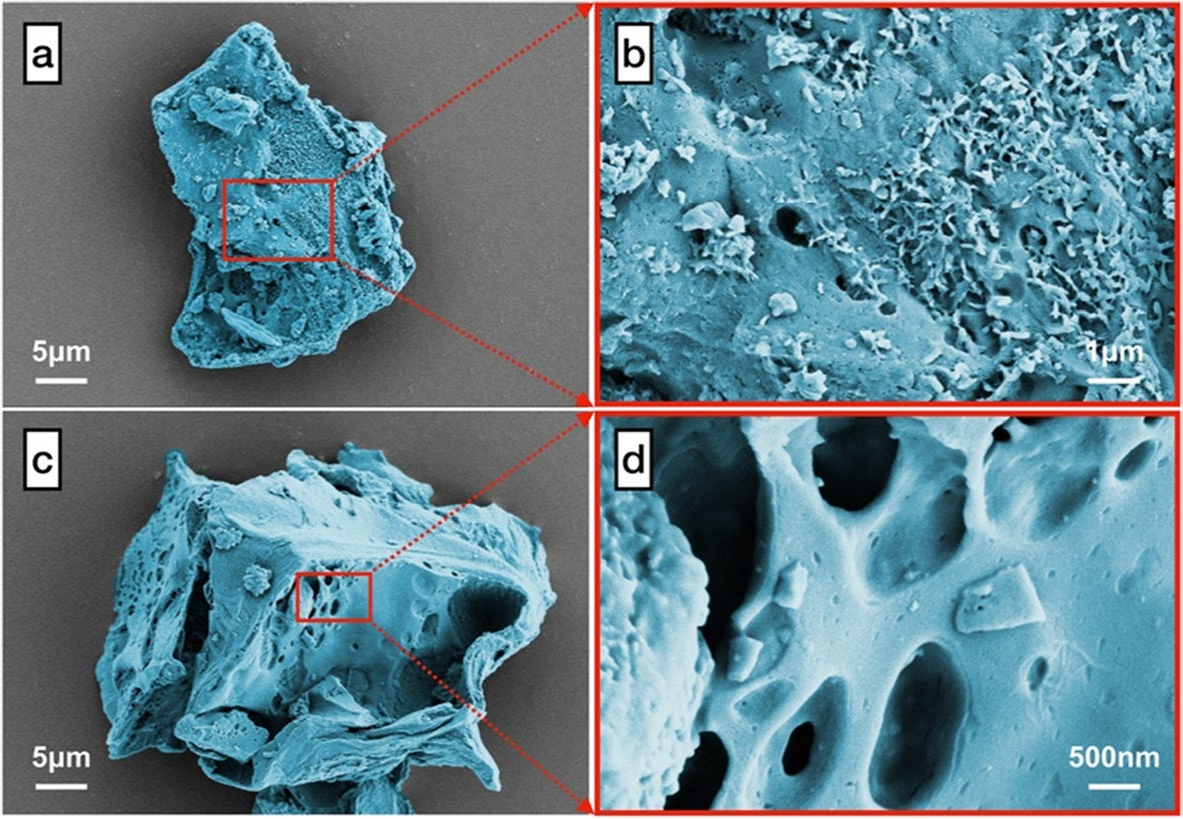
False-color SEM images of SCGs (SCGs particles were colored with blue)

The frequency sweep curves of complex shear modulus (G*) and phase angle (*δ*) for the SCGs modified binders together with the neat binder were determined in Fig. 6 (a) and (b) . It can be noticed that the adoption of 1% and 3% SCGs contributes to an shift of complex shear modulus values across all frequencies compared to the control binder, which indicates that the undamaged stiffness of modified binders has been improved. Furthermore, the binder with 3% SCGs exhibits the highest G* values at low frequencies, confirming that SCGs are effective as the modifier in improving the shearing stress-resistant ability of asphalt binders. However, such increment of G* values will not be strengthened further along with the increased dosage of SCGs. The binder with 5% SCGs shows slightly lower complex modulus than that of the control. This is due to the fact that the agglomeration of excessive SCGs offsets the improved stiffness of modified binders. From the frequency sweep results of phase angle demonstrated in Fig. 6, the *δ* values of modified binders are reduced in comparison with the control sample across all frequencies, since the incorporation of SCGs increased the elastic recovery performance of binders. As a type of reinforced and porous solid waste (shown in Fig. 7), SCGs are able to absorb oil and resin from asphalt, thus the proportion of elastic components has relatively increased with the fluidity of binders decreased, which leads to the decline of phase angle. Besides, it is worth mentioning that scarcely does the increasing content of SCGs in binders influence the variation of the phase angle values, whereas 1% and 3% SCG-incorporated binders still present lower *δ* values among the binders with different addition of SCGs modified than the control sample. Also, the outcomes from the frequency sweep curves are coherent with the results achieved from the rotational viscosity and rutting factors.

### High-temperature performance

**Fig. 8:**
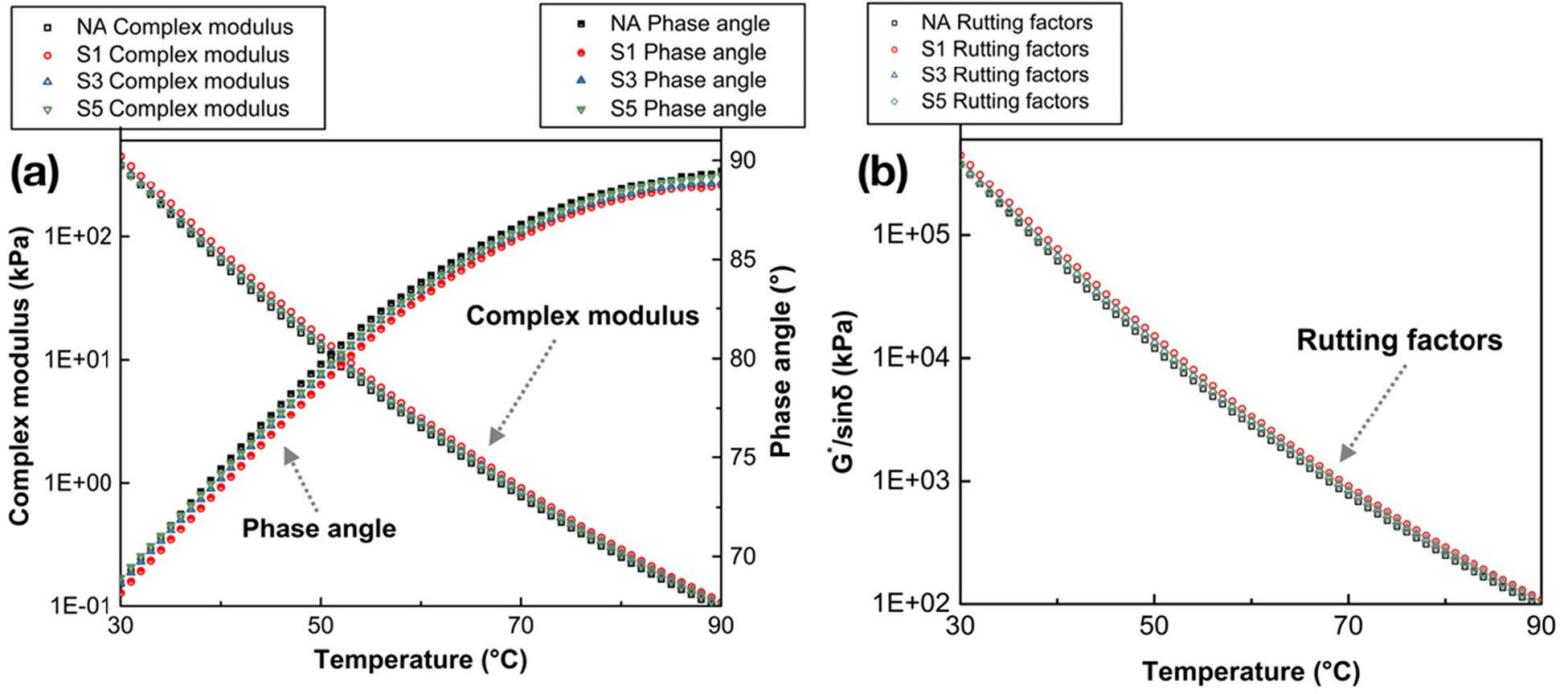
The temperature sweep results of SCGs modified binders and NA binder, (a) complex shear modulus and phase angle; (b) the rutting factors (G*/sinδ) of SCGs modified binders and NA binder

**Fig. 9:**
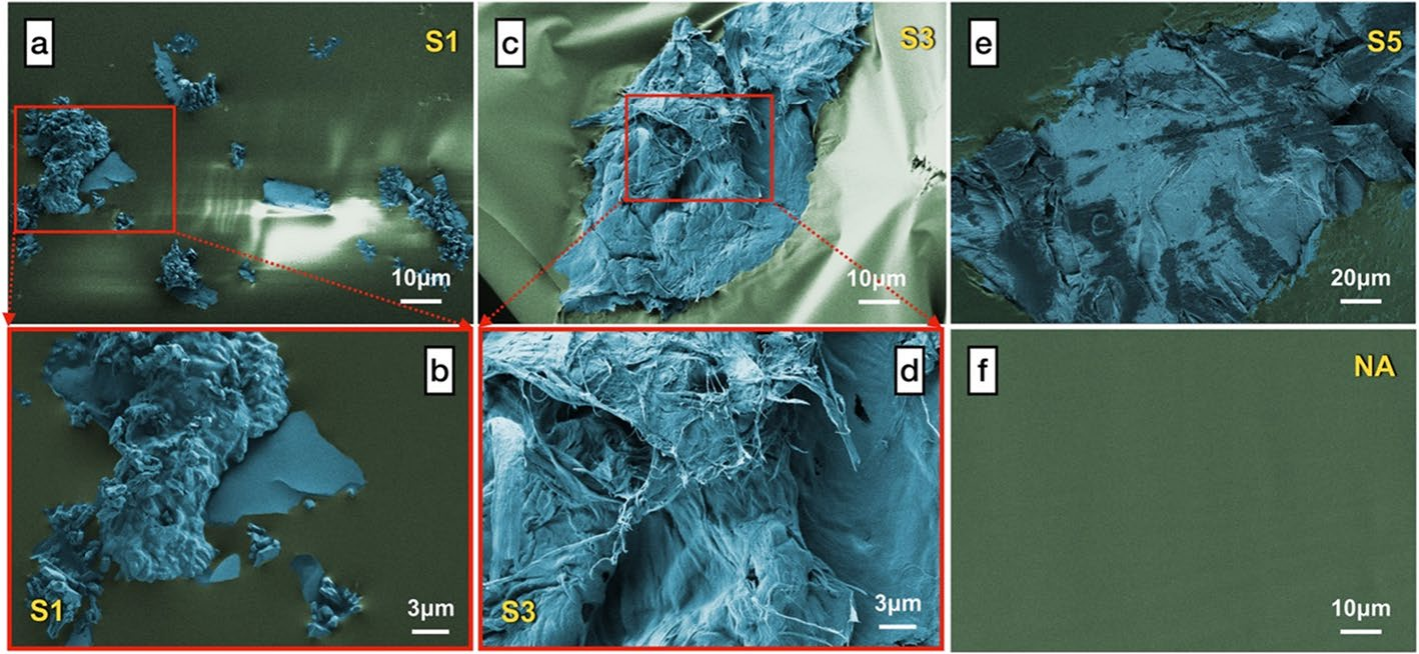
False-color SEM images of tested asphalt binders (the neat binders with 60/80 penetration grade were colored with green; SCGs particles were colored with blue), (**a**) ~ (**b**) the modified binder with 1% SCGs; (**c**) ~ (**d**) the modified binder with 3% SCGs; (**e**) the modified binder with 5% SCGs; (**f**) the neat asphalt binder

Complex shear modulus (G*) and phase angle (*δ*) were summarized by using DSR with the temperature ranging from 30 °C to 90 °C. The temperature sweep results of SCGs modified binders and NA binder are presented in Fig. 8(a); meanwhile, the rutting factors (G*/sinδ), which implies the high-temperature rutting resistance of specimens, are also shown in Fig. 8(b). From Fig. 8(a), the complex shear moduli of binders exhibit a declining trend with the sweeping temperature rising, while the phase angle (Fig. 8(a)) increases owing to the decreased elasticity and increased viscosity of asphalt binder. After being incorporated by SCGs, the complex shear moduli of binders are slightly increased, while the phase angles are in decline compared with the neat binder, which illustrates their better responses to the applied force. The results also indicate that the SCGs modified binders have the enhanced ability to resist high-temperature shearing stress. Nonetheless, it is remarkably to mention that this increased resistance to shearing stress is rather limited. The rutting factors of binders were calculated and shown in Fig. 8(b). Compared with the neat binder, the modified binders exhibit a bit higher G*/sinδ values at the same temperature, but these changes can be negligible. It implies that the adoption of SCGs almost has no difference in the rutting resistance of asphalt binders, except for the binders with 1% SCGs exhibiting relatively better performance. This enhancement can be attributed to the increased modulus and lower phase angle of the modified asphalt binders. With SCGs modified, the viscous components in asphalt are decreased and the light components are inclined to absorb into the porous SCG particles (Fig. 9), which has been discussed in the following **Sections 3.6** and **3.7**.

### Creep and recovery behavior

**Fig. 10:**
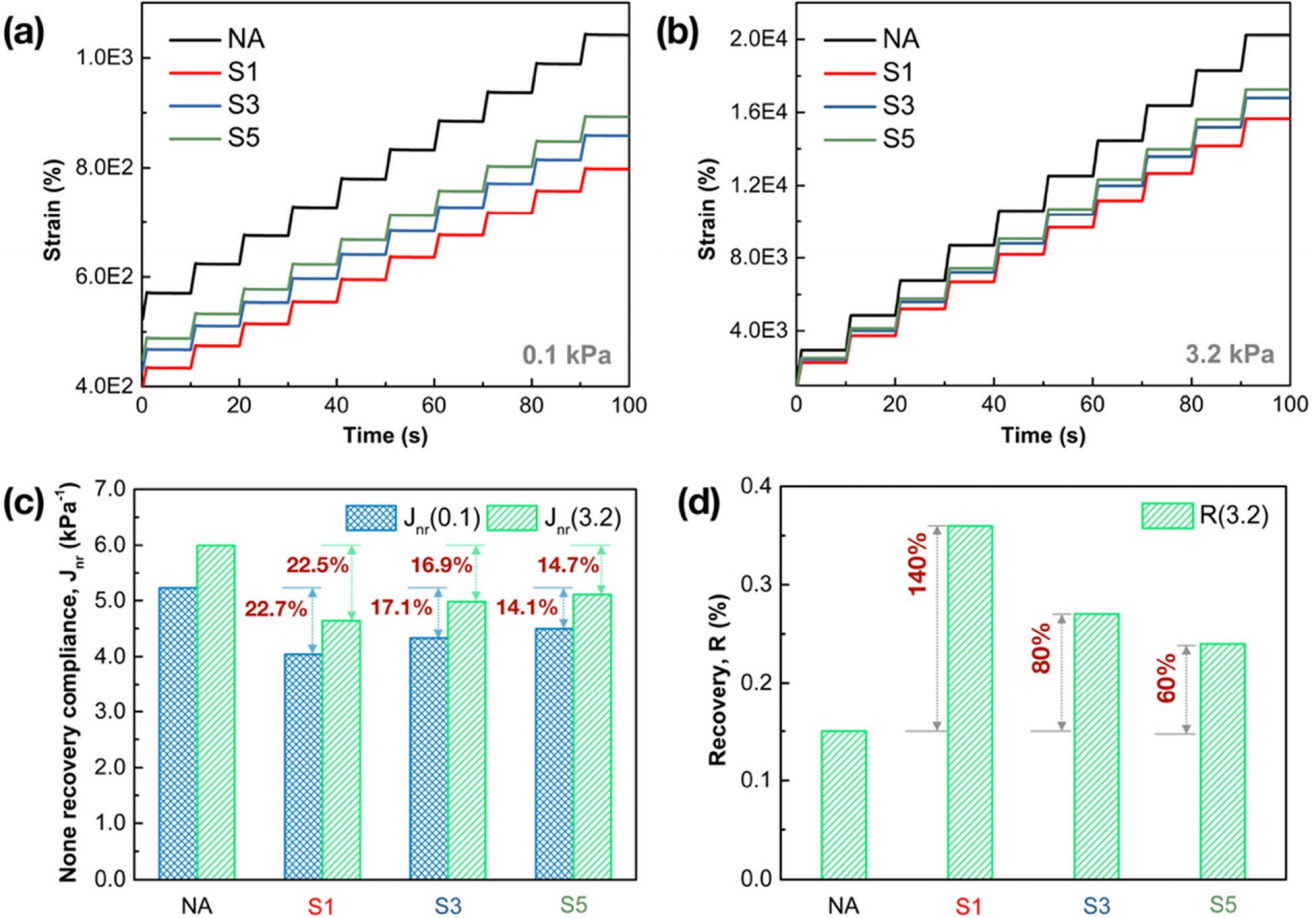
The time-strain curves of MSCR results for SCGs modified binders and NA binder at different stress levels, (**a**) 0.1 kPa; (**b**) 3.2 kPa; (**c**) the non-recoverable compliance (*J*_*nr*_) values at 0.1 kPa and 3.2 kPa; (**d**) the recovery (*R*) values at 3.2 kPa

The creep and recovery behavior of asphalt binders investigated by the MSCR test is also essential to demonstrate the permanent deformation resistance under high-temperature performance. The time-strain curves of MSCR results are shown in Fig. 10(a) and (b) at two creep-recovery stress levels of 0.1 kPa and 3.2 kPa respectively. It can be seen that the accumulated strains of modified asphalt binders are remarkably reduced owing to the incorporation of SCGs at both loading levels, among which the binder with 1% SCGs exhibits the lowest accumulated strains, and then followed by 3% and 5%. This provides a strong indication that SCGs addition is beneficial to improve the permanent deformation resistance of binders, in that the adoption of SCGs works by effectively increasing the viscosity of binder, which complies with our discussion above. Fig. 10(a) and (b) also illustrate that a dose of 1% can be considered as the optimal incorporated content of SCGs, whereas the improvement of shear strain responses will be restricted or even weakened as the dosage rises to 3% or 5%.

Two parameters from MSCR tests, non-recoverable compliance (*J*_*nr*_) values and recovery (*R*) values are presented in Fig. 10(c) and (d) at different loading levels respectively. *J*_*nr*_ is considered as the predominant parameter to evaluate the rutting performance of asphalt binders. From the results shown in Fig. 10(c), the adoption of SCGs modified generally leads to the lower *J*_*nr*_(0.1) and *J*_*nr*_(3.2) values compared to the neat binder, demonstrating the rutting resistance has been greatly improved. Besides, the binder with 1% SCGs exhibits the best rutting resistance, since the values of *J*_*nr*_(0.1) and *J*_*nr*_(3.2) exhibit 22.7% and 22.5% lower than that of the neat binder. The result indicates that 1% SCGs modified binder was expected to have the best resistance to deformation. Nevertheless, this enhancement is offset by the increase in SCGs content as well. The increasing SCGs modifier percentage of 3% or 5% is found to induce a lower rutting potential for binders than that of the 1% SCGs modified binder, though the modified binders still present higher rutting performance than the control. This is mainly due to the oil fractions in SCGs weaken the physical reinforcement of SCGs modification. The increased organic groups of modified binders can be verified from the FT-IR results (as shown in Fig. 4) as compared to that of the control.

Figure 10(d) displays the *R*(3.2) values of different asphalt binders, which is typically used to distinguish the delayed elastic behavior of binder. After modified by SCGs, the binders exhibit higher *R*(3.2) values than that of the neat binder during the process of creep-recovery loading sequences; meanwhile, the binder with 1% SCGs ranks the highest *R*(3.2) value, which has increased by 140% than the control; then followed by the binders with 3% SCGs and 5% SCGs, which has increased by 80% and 60%, respectively. It remarks that this increment of *R*(3.2) values is mainly attributed to the improved elasticity of binders. With the addition of SCGs, the contents of light components have been reduced and the colloid structure of asphalt binder becomes more stable, thus improving the elasticity of binders. Meanwhile, the appropriate addition of SCGs (1%) is regarded as the effective condition to promote the recovery performance of asphalt binders. Owing to the agglomeration of excessive SCGs, 3% ~ 5% SCGs content exterts a negative restriction on the improvement of *R*(3.2) value. The reason was that the agglomeration of SCGs may exert passive effects on the creep and recovery responses of asphalt binder. But it is worth mentioning that the resistance to deformation and the recoverability of modified binders are still better as compared to the neat asphalt, illustrating the effectiveness of SCGs modification.

### Low-temperature thermal stability

**Fig. 11:**
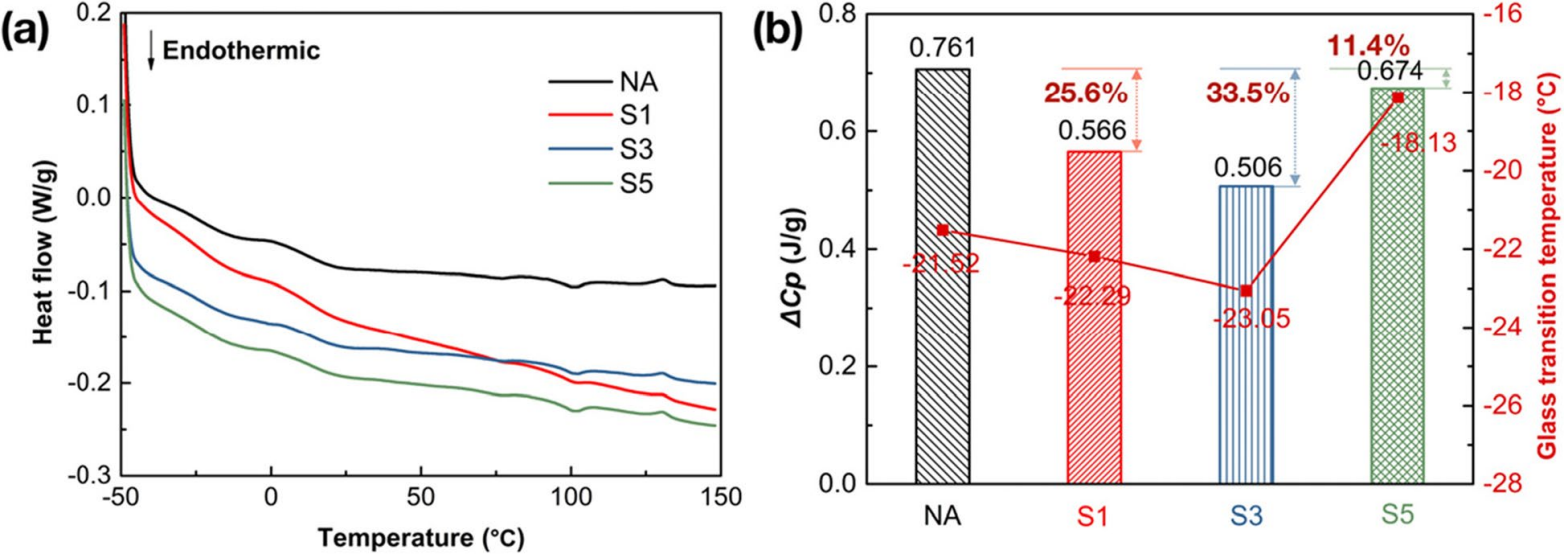
Low-temperature performance of tested binders performed by DSC analysis, **a** DSC curves; **b** the glass transition temperature (*T*_*g*_) and the endothermic enthalpy (*△cp*) for SCGs modified binders and NA binder from DSC curves

DSC was performed to investigate the temperature dependent-behavior of the different binders and the results are summarized in Fig. 11(a) and (b). From Fig. 11(b), the incorporation of SCGs contributes to the continuous reduction of the glass transition temperature (*T*_*g*_) values at low temperature for the asphalt binders as the percentage increases from 1% to 3%; whereas the *T*_*g*_ value increases from − 21.52 °C to − 18.13 °C when the dosage comes up to 5%. This is because the addition of SCGs leads to the increased viscoelasticity to elasticity in the modified asphalt binders, which can be supported by the results obtained from the previous rheology analysis. Besides, the addition of SCGs at an optimal dosage is more effective to decelerate the phase change of neat binders, thus reducing the vitrification transformation; moreover, it is worth mentioning that the binder with 3% SCGs exhibits the lowest *T*_*g*_ value, of which the low-temperature stability has been evidently improved. Consistent with our discussion above, the decline of phase transition temperature at low temperature can be observed with the adoption of overdosing modifier.

The thermal transition energy values from DSC curves characterizes the agglomeration state of materials. Typically a lower endothermic enthalpy (*△cp*) value represents higher thermal stability. As shown in Fig. 11(b), the *△cp* values determined from the process of glass transition are generally considered as the prime parameter to evaluate the thermal behavior of asphalt molecules. Similar to the discussion above of *T*_*g*_, the *△cp* values at low temperature of modified binders are decreased with the increased content of SCGs from 1% to 3%, which indicates they become more stable with SCGs modified. Asphalt binder frequently presents a weak connection between molecules due to the low molecular weight (no more than 6000 in general) [41]. The porous structure of SCGs (Fig. 7) can absorb more light components in asphalt and the movements of molecules can be effectively restricted with the incorporation of SCGs, indicating positive effects on enhanced low-temperature stability. Meanwhile, it can also be detected from Fig. 11(b) that the modified binders possess ideal low-temperature stability with the percentage of SCGs no more than 3%, especially for a dosage of 3%, which is 33.5% lower than that of the control. Notwithstanding, the low-temperature stability of the binder with 5% SCGs is inferior to the control with regard to the reduced *△cp* value for modified binders. This can be attributed to the increased amount of the isolated pores in SCGs when the dosage of SCGs is up to 5% (as discussed in **Section 3.7**), thus leading to the degradation of low-temperature stability.

### Micro-morphology and structure

Figs. 7 and 9 present the micro-morphology of SCGs and the tested binders from SEM images respectively. For better visual presentation, false-colored SEM images have been adopted to differentiate SCGs and asphalt binders. It can be clearly noticed from Fig. 7(a)–(d) that SCGs exhibit the corrugated and porous surface with irregular shapes as a result of extraction, thus they provide the enclosing space as the reinforcement modifier for accommodating asphalt binder through the high-speed shearing process. Additionally, the SEM images of modified binders along with the control are shown in Fig. 9(a)–(e). The distribution of SCGs in S1 is shown in Fig. 9(a), and it can be observed from Fig. 9(a)–(b) that SCGs are successfully immersed into the asphalt binders compared with the control sample shown in Fig. 9(f). With the incorporation of SCGs, the light components in asphalt are more inclined to absorb into SCGs in terms of their porous structure, leading to improved stability and elasticity for asphalt binders. Meanwhile, the rough and jagged surface of SCGs can be afforded for enhanced interfacial compatibility between SCGs particles and binders. The cross-sections of SCGs in modified asphalt binders are presented in Fig. 9(c) and Fig. 9(e); also, the details about the cross-section of SCGs in S3 are displayed in Fig. 9(d). As the reinforcement phase, SCG particles exhibit corrugated and crude fiber-like structure, which provides the possibility to enhance rheological, mechanical and physical properties. Beneficial from the improved bonding and mechanical strength, the modified binders have better performance to resist deformation as discussed above. However, this phenomenon does not last until the continuity of binders has been evidently blocked or even wrecked with the excessive addition of SCGs. The increased isolated pores within SCG particles exert negative effects when they are not filled with the binder, and thus offset the reinforcement from the modification of SCGs. These micro-morphology features can be reasonably excused for the degradation of enhanced properties as the discussion above.

## Cost analysis

**Fig. 12:**
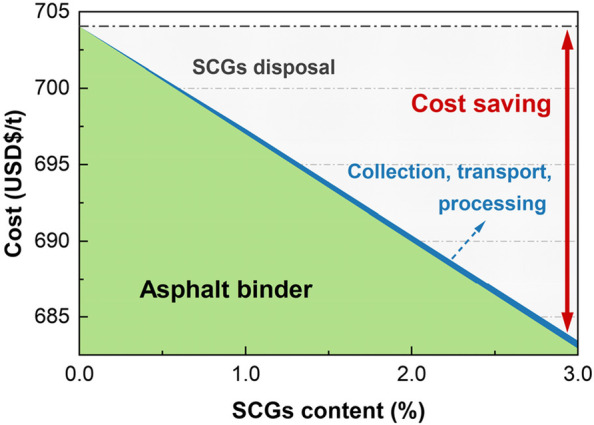
Material-related costs of SCGs modified asphalt

The cost analysis has been an important segment for the circular economy and cleaner production during the cycle of solid waste utilization. A simple calculation has been investigated to assess the related cost for the produced asphalt with the increased SCGs content ranging from 0% to 3%, and the results were summarized in Fig. 12. According to the data from Martins, et al. [42], the unit price of materials and operations for producing asphalt binder costs about 704 USD($) per ton(t), which is the major expense in the operation of asphalt mixture. With the incorporation of 1% ~ 3% SCGs, the net unit price of modified asphalt per ton can be reduced by 7.04 $/t ~ 21.12 $/t. However, it is difficult to determine the actual viable costs of the collection, transport, and processing of SCGs; also, there still exists a gap in handling SCGs for commercial and industrial promotion by commercial organizations. In this work, a possible solution for the cost analysis proposed by Saberian et al. [39] was adopted to estimate the associated expenses of solid waste materials with their collection, transport and processing, which was determined as 35 $/t in accordance with Sustainability Victoria [43]. After excluding the extra cost from the collection, transport, and processing, the modification of 1% ~ 3% SCGs in asphalt binder results in a cost saving of 6.69 $/t ~ 20.60 $/t hereby. Moreover, it can be expected that the pavements newly constructed would reach 25 million kilometers by 2050 worldwide [44, 45]. Plus about 5485.3 million tons of asphalt were produced in 32 countries from 2008 to 2015 [46, 47], and the price of asphalt continues to increase [42, 48]. Ergo, SCGs modified asphalt is of great expectation to achieve the cost-effective utilization of solid waste with the augmented financial saving, and the logistics and transportation costs are almost negligible as compared to the cost saving.

## Conclusion and remarks

In this work, SCGs were adopted as a cost-effective modifying filler to achieve dual objectives, including sustainable management of bio-waste residual and targeted enhancement of asphalt binders. The results demonstrated the modification process was mainly based on physical reinforcing between asphalt binders and SCGs. SEM images showed that SCGs filler has been sufficiently immersed into the neat asphalt binders. With the appropriate addition of SCGs, moreover, the modified binders exhibit better low-temperature stability and improved high-temperature performance as compared to the control. The incorporation of SCGs contributes to the reduced *T*_*g*_ and *△cp* values with percentages of 1% and 3%, which accordingly improves the low-temperature stability of binders. It is indicated that the light components in asphalt are more inclined to absorb into SCGs in terms of their porous structure.

However, the excessively increasing content of SCGs exerts negative effects on the practical properties of asphalt binders, which is caused by the agglomeration of excessive SCGs in the asphalt. Besides, the increased isolated pores in SCGs that are not filled with asphalt exhibit adverse effects on the properties of asphalt as well. Hence, there still requires necessary selection for content before applied.

This study aims to provide a sustainable, facile but effective approach for the utilization of bio-waste residual from the coffee industry. It is remarkable to conclude that SCGs have potential prospects as a modifier in sustainable pavements, meanwhile, the increasingly huge amount of pavement construction has thoroughly created such marketing possibilities for their large-scale application. In addition, more efforts should be made for the research on how the particle sizes and impurities mixing of SCGs affect the performance of asphalt. Also, the applied effects and long-term performance of asphalt mixture should be considered in the further investigation. It is noteworthy that the cost of pretreatments and reutilizing approaches still needs to be comprehensively estimated during the life cycle of SCGs for future study as well.

The authors gratefully acknowledge the financial supports provided by the National Natural Science Foundation of China (51,878,480, 52,078,369), and the Fundamental Research Funds for the Central Universities. The authors also would like to acknowledge Professor Zhihua Zhong (Academician of the Chinese Academy of Engineering) and the financial supports from Intelligent Vehicle Stream, TJU-THU-HUN Collaborative Innovation Institute of Science & Technology, Qingdao International Academician Park Co. Ltd. The first author is supported by the scholarship from the China Scholarship Council (CSC, No. 202206260066), which is sincerely appreciated. The authors also want to give their sincere thanks to the novel conception from Hongmi Jiang at Shanghai Pinghe Bilingual School for the experiments of this work.

## References

[1] Mussatto, S. I., Machado, E. M. S., Martins, S., & Teixeira, J. A. (2011). Production, composition, and application of coffee and its industrial residues. *Food and Bioprocess Technology, 4*(5), 661–672.

[2] Marescotti, A., & Belletti, G. (2016). Differentiation strategies in coffee global value chains through reference to territorial origin in Latin American countries. *Culture and History Digital Journal, 5*(1).

[3] Crossley, O. P., Thorpe, R. B., Peus, D., & Lee, J. (2020). Phosphorus recovery from process waste water made by the hydrothermal carbonisation of spent coffee grounds. *Bioresource Technology, 301*. 10.1016/j.biortech.2019.122664 31931334

[4] ICO. (2020). *Coffee Development Report*. ICO.

[5] Kim, J., Kim, H., Baek, G., & Lee, C. (2017). Anaerobic co-digestion of spent coffee grounds with different waste feedstocks for biogas production. *Waste Management, 60*, 322–328. 27751681 10.1016/j.wasman.2016.10.015

[6] Janissen, B., & Huynh, T. (2018). Chemical composition and value-adding applications of coffee industry by-products: A review. *Resources Conservation and Recycling, 128*, 110–117.

[7] Yi, X. (2021). Shanghai found to be city with most coffee shops in the world, *China Daily*. http://www.chinadaily.com.cn/a/202103/30/WS6062d885a31024ad0bab2906.html.

[8] Chunyuan, Z. (2021). Shanghai leads world in coffee stores. *Eastday*.

[9] Yicai, S. C. (2021). Consumption index report. *Yicai Global*.

[10] Mata, T. M., Martins, A. A., & Caetano, N. S. (2018). Bio-refinery approach for spent coffee grounds valorization. *Bioresource Technology, 247*, 1077–1084. 28969966 10.1016/j.biortech.2017.09.106

[11] Acevedo, F., Rubilar, M., Scheuermann, E., Cancino, B., Uquiche, E., Garces, M., Inostroza, K., & Shene, C. (2013). Spent coffee grounds as a renewable source of bioactive compounds. *Journal of Biobased Materials and Bioenergy, 7*(3), 420–428.

[12] Obruca, S., Benesova, P., Petrik, S., Kucera, D., & Marova, I. (2014). Biotechnological conversion of spent coffee grounds into polyhydroxyalkanoates. *New Biotechnology, 31*, S39–S40. 10.1016/j.nbt.2015.02.008 25721970

[13] Shang, Y. F., Xu, J. L., Lee, W. J., & Um, B. H. (2017). Antioxidative polyphenolics obtained from spent coffee grounds by pressurized liquid extraction. *African Journal of Botany, 109*, 75–80.

[14] Hudeckova, H., Neureiter, M., Obruca, S., Fruhauf, S., & Marova, I. (2018). Biotechnological conversion of spent coffee grounds into lactic acid. *Letters in Applied Microbiology, 66*(4), 306–312. 29330879 10.1111/lam.12849

[15] Kante, K., Nieto-Delgado, C., Rene Rangel-Mendez, J., & Bandosz, T. J. (2012). Spent coffee-based activated carbon: Specific surface features and their importance for H2S separation process. *Journal of Hazardous Materials, 201*, 141–147. 22154120 10.1016/j.jhazmat.2011.11.053

[16] Kim, M.-S., Min, H.-G., Koo, N., Park, J., Lee, S.-H., Bak, G.-I., & Kim, J.-G. (2014). The effectiveness of spent coffee grounds and its biochar on the amelioration of heavy metals-contaminated water and soil using chemical and biological assessments. *Journal of Environmental Management, 146*, 124–130. 25242543 10.1016/j.jenvman.2014.07.001

[17] Kondamudi, N., Mohapatra, S. K., & Misra, M. (2008). Spent coffee grounds as a versatile source of green energy. *Journal of Agricultural and Food Chemistry, 56*(24), 11757–11760. 19053356 10.1021/jf802487s

[18] Caetano, N. S., Silva, V. F. M., Melo, A. C., Martins, A. A., & Mata, T. M. (2014). Spent coffee grounds for biodiesel production and other applications. *Clean Technologies and Environmental Policy, 16*(7), 1423–1430.

[19] Jalkh, R., El-Rassy, H., Chehab, G. R., & Abiad, M. G. (2018). Assessment of the Physico-chemical properties of waste cooking oil and spent coffee grounds oil for potential use as asphalt binder rejuvenators. *Waste and Biomass Valorization, 9*(11), 2125–2132.

[20] Battista, F., Zanzoni, S., Strazzera, G., Andreolli, M., & Bolzonella, D. (2020). The cascade biorefinery approach for the valorization of the spent coffee grounds. *Renewable Energy, 157*, 1203–1211.

[21] Jiang, Z., Li, W., & Yuan, Z. (2015). Influence of mineral additives and environmental conditions on the self-healing capabilities of cementitious materials. *Cement and Concrete Composites, 57*, 116–127.

[22] Li, W., Dong, B., Yang, Z., Xu, J., Chen, Q., Li, H., Xing, F., & Jiang, Z. (2018). Recent advances in intrinsic self-healing cementitious materials. *Advanced Materials, 30*(17). 10.1002/adma.201705679 29577476

[23] Yang, Q., Li, C., Ren, Q., & Jiang, Z. (2021). Properties and microstructure of CO2 activated binder produced by recycling phosphorous slag. *Construction and Building Materials, 282*, 122698.

[24] Qin, L., Gao, X., Su, A., & Li, Q. (2021). Effect of carbonation curing on sulfate resistance of cement-coal gangue paste. *Journal of Cleaner Production, 278*, 123897.

[25] Leng, Z., Padhan, R. K., & Sreeram, A. (2018). Production of a sustainable paving material through chemical recycling of waste PET into crumb rubber modified asphalt. *Journal of Cleaner Production, 180*, 682–688.

[26] Yu, H. Y., Leng, Z., Zhou, Z. Y., Shih, K. M., Xiao, F. P., & Gao, Z. M. (2017). Optimization of preparation procedure of liquid warm mix additive modified asphalt rubber. *Journal of Cleaner Production, 141*, 336–345.

[27] Eliche-Quesada, D., Martínez-García, C., Martínez-Cartas, M. L., Cotes-Palomino, M. T., Pérez-Villarejo, L., Cruz-Pérez, N., & Corpas-Iglesias, F. A. (2011). The use of different forms of waste in the manufacture of ceramic bricks. *Applied Clay Science, 52*(3), 270–276.

[28] Eliche-Quesada, D., Perez-Villarejo, L., Iglesias-Godino, F. J., Martinez-Garcia, C., & Corpas-Iglesias, F. A. (2011). Incorporation of coffee grounds into clay brick production. *Advances in Applied Ceramics, 110*(4), 225–232.

[29] Munoz Velasco, P., Mendivil, M. A., Morales, M. P., & Munoz, L. (2016). Eco-fired clay bricks made by adding spent coffee grounds: A sustainable way to improve buildings insulation. *Materials and Structures, 49*(1–2), 641–650.

[30] A. Lachheb, A. Allouhi, M. El Marhoune, R. Saadani, T. Kousksou, A. Jamil, M. Rahmoune, O. Oussouaddi, Thermal insulation improvement in construction materials by adding spent coffee grounds:. An experimental and simulation study, Journal of Cleaner Production 209 (2019) 1411–1419.

[31] da Fonseca, B. S., Vilao, A., Galhano, C., & Simao, J. A. R. (2014). Reusing coffee waste in manufacture of ceramics for construction. *Advances in Applied Ceramics, 113*(3), 159–166.

[32] Arulrajah, A., Maghoolpilehrood, F., Disfani, M. M., & Horpibulsuk, S. (2014). Spent coffee grounds as a non-structural embankment fill material: Engineering and environmental considerations. *Journal of Cleaner Production, 72*, 181–186.

[33] Arulrajah, A., Kua, T.-A., Phetchuay, C., Horpibulsuk, S., Mahghoolpilehrood, F., & Disfani, M. M. (2016). Spent coffee grounds-Fly ash Geopolymer used as an embankment structural fill material. *Journal of Materials in Civil Engineering, 28*(5).

[34] Kua, T.-A., Arulrajah, A., Horpibulsuk, S., Du, Y.-J., & Shen, S.-L. (2016). Strength assessment of spent coffee grounds-geopolymer cement utilizing slag and fly ash precursors. *Construction and Building Materials , 115*, 565–575.

[35] Kua, T.-A., Arulrajah, A., Horpibulsuk, S., Du, Y.-J., & Suksiripattanapong, C. (2017). Engineering and environmental evaluation of spent coffee grounds stabilized with industrial by-products as a road subgrade material. *Clean Technologies and Environmental Policy, 19*(1), 63–75.

[36] Goh, B. H. H., Ong, H. C., Chong, C. T., Chen, W.-H., Leong, K. Y., Tan, S. X., & Lee, X. J. (2020). Ultrasonic assisted oil extraction and biodiesel synthesis of spent coffee ground. *Fuel, 261*, 116121.

[37] Chun, Y., Ko, Y. G., Do, T., Jung, Y., Kim, S. W., & Choi, U. S. (2019). Spent coffee grounds: Massively supplied carbohydrate polymer applicable to electrorheology. *Colloids and Surfaces A: Physicochemical and Engineering Aspects, 562*, 392–401.

[38] D.J. Lyman, R. Benck, S. Dell, S. Merle, J. Murray-Wijelath, FTIR-ATR analysis of brewed coffee: Effect of roasting conditions, Journal of Agricultural and Food Chemistry 51(11) (2003) 3268-3272. 10.1021/jf0209793 12744653

[39] Saberian, M., Li, J., Donnoli, A., Bonderenko, E., Oliva, P., Gill, B., Lockrey, S., & Siddique, R. (2021). Recycling of spent coffee grounds in construction materials: A review. *Journal of Cleaner Production, 289*, 125837.

[40] Wang, P. E. Y., Wen, Y., Zhao, K., Chong, D., & Wong, A. S. T. (2014). Evolution and locational variation of asphalt binder aging in long-life hot-mix asphalt pavements. *Construction and Building Materials, 68*, 172–182.

[41] Jin, X., Guo, N., You, Z., Wang, L., Wen, Y., & Tan, Y. (2020). Rheological properties and micro-characteristics of polyurethane composite modified asphalt. *Construction and Building Materials*, 234.

[42] Zaumanis, M., Mallick, R. B., & Frank, R. (2014). 100% recycled hot mix asphalt: A review and analysis. *Resources Conservation and Recycling, 92*, 230–245.

[43] Sustainability, V. (2014). Market summary – Recycled glass. *Sustainability Victoria*, 1–5.

[44] Laurance, W. F., Clements, G. R., Sloan, S., O'Connell, C. S., Mueller, N. D., Goosem, M., Venter, O., Edwards, D. P., Phalan, B., Balmford, A., Van Der Ree, R., & Arrea, I. B. (2014). A global strategy for road building (vol 513, pg 229, 2014). *Nature, 514*(7521), 262–262. 10.1038/nature13717 25162528

[45] Plati, C. (2019). Sustainability factors in pavement materials, design, and preservation strategies: A literature review. *Construction and Building Materials, 211*, 539–555.

[46] Jahanbakhsh, H., Karimi, M. M., Naseri, H., & Moghadas Nejad, F. (2020). Sustainable asphalt concrete containing high reclaimed asphalt pavements and recycling agents: Performance assessment, cost analysis, and environmental impact. *Journal of Cleaner Production, 244*.

[47] EAPA, Brussels, Belgium, (2015). https://scholar.google.com/scholar_lookup?title=Brussels%2C%20Belgium&author=EAPA&publication_year=2015.

[48] Pennsylvania Asphalt Pavement Association. Harrisburg: Inex; (2021). https://www.pa-asphalt.org/images/June_2022.pdf.

